# School-Provided Meals and the Prevention of Childhood Obesity: A Small part of a Very Important Story

**DOI:** 10.1007/s13679-025-00635-x

**Published:** 2025-05-15

**Authors:** Danielle Gallegos, Alexandra Manson, Helen Anna Vidgen, Rebecca Byrne, Brittany J. Johnson

**Affiliations:** 1 School of Exercise and Nutrition Sciences, Faculty of Health, Queensland University of Technology (QUT), Victoria Park Rd, Kelvin Grove, Brisbane City, QLD 4059 Australia; 2 Centre for Childhood Nutrition Research, Faculty of Health, Queensland University of Technology (QUT), 61 Graham St, South Brisbane, Brisbane City, QLD 4101 Australia; 3 College of Nursing and Health Sciences, Flinders University, Caring Futures Institute, Sturt Road, Bedford Park, SA 5042 Australia

**Keywords:** School meals, Nutrition, Whole of school approach, Health-promoting school, Food and nutrition security

## Abstract

**Purpose of Review:**

To present the evidence base in support of high-income country investment in universal school-provided meals (SPMs) for the purposes of optimising child health and wellbeing, including obesity prevention.

**Recent findings:**

Many countries provide some form of SPMs. Models (universal, free; targeted; subsidised) vary globally, however optimal growth and development of children as a potential outcome is a consistent feature. SPMs can positively impact diet quality, household food and nutrition security and potentially weight status but is dependent on the model. Universal school meals offered as part of whole-of-school approaches appear to be most effective in optimising children’s growth and development. Critical elements for successful SPMs include being underpinned by enforceable nutrition and sustainability standards, offered in ways that are stigma-free, being embedded within a whole-school approach and conceptualising SPMS as part of transformative food systems.

**Summary:**

Weight status is only one of many potential outcomes of SPMs. Implementing universal SPMs is a triple duty action that can address the global syndemic of obesity, undernutrition and climate change. Attention needs to be paid to the model of implementation and key principles for success.

## Introduction

School-provided meals (SPMs) are supplied to students, and sometimes staff, in the form of breakfast and/or lunch and/or snacks (for example, fruit or vegetables) by the school and eaten in that setting. Many countries provide some form of SPMs reaching one in four primary and secondary school aged children [1]. Given that most children from about four years of age to at least 12, and in many countries up to 16 years, attend compulsory school, the provision of food is an opportunity to influence the health and wellbeing of children during critical growth periods of growth and development [2]. The World Health Organization (WHO), World Food Programme and Food and Agriculture Organization support their implementation as one of the world’s most comprehensive global safety nets [3–5]. However, SPMs differ extensively in their purpose, composition, delivery, universality, cost and model of implementation. Despite this variability, optimal growth and development of children is a consistently identified outcome of SPMs. Low-income countries focus on malnutrition presenting as poor diet quality and underweight, while high-income countries are more likely to focus on malnutrition presenting as poor diet quality and overweight/obesity [3]. Middle-income countries are in a bit of a quandary with SPMs needing to address the double and triple burden of disease often in contexts where the nutrition transition is occurring rapidly [6].

Implementation models vary between countries and within countries [7]. SPMs can be provided universally or to selected children, they can be fully or partially subsidised by government, require co-payments or be fully funded by caregivers [6, 7]. Provision models include outsourced food service providers, to in-school preparation (from either fresh or pre-prepared foods) with or without circular economy models that connect to local growers and producers and minimise environmental impacts [7, 8]. SPMs may form part of a school curriculum extending classroom lessons on culture, health, sustainability, and social relationships, that can be delivered in spaces and times outside the classroom [9]. Involvement of nutrition professionals in the planning, implementation, monitoring and evaluation of SPMs, also varies, from no involvement at all, to joint delivery with a local health authority. All these factors impact on how successful SPMs are in influencing child physical, psychological, social and academic outcomes [7].

The global rise in childhood overweight and obesity has focussed attention on universal strategies that could be used to prevent and manage the condition [10]. In this context, the WHO has cited schools as one of the key settings for action [11]. The effectiveness of SPMs in the prevention of overweight and obesity, however, needs to consider variation in implementation models. More importantly, the optimal implementation of SPMs represents an opportunity for benefits extending far beyond this single chronic disease risk factor. Obesity prevalence is often presented as an outcome measure of child nutrition in national and more local jurisdictional policy with associated monitoring and surveillance, in part because it is an easily quantified and measurable health indicator that can be linked to tangible economic benefits that can be easily communicated. If framing SPMs against this health outcome, however, a holistic approach to prevention should be referenced. This includes acknowledgement of the global syndemic of obesity, undernutrition and climate change; the inequitable distribution of health risk; and the contribution of social, commercial and political determinants of health, particularly the financialization of food [12]. It is accepted that to halt or reverse the rise in the prevalence of overweight and obesity, significant food system reform is required. SPMs present an opportunity to re-orient the agrifood system to being healthier, fairer and more sustainable at an achievable scale.

The United Nations General Assembly has recently extended the Decade of Action on Nutrition in recognition of ongoing importance of efforts to address malnutrition in all its forms. SPMs are identified as a key strategy in the achievement of this [13].

This perspectives paper is a critical narrative review of the current evidence of the role of SPMs in obesity prevention; how SPMs can potentially contribute to overall child health and wellbeing via various pathways; and the role of SPMs in addressing the triple threat of the global syndemic. It concludes with identifying the essential principles of SPMs programs, using examples of effective implementation throughout the world, that can be used to support investment in SPMs within high-income country contexts.

## Current Evidence: SPMs and Obesity

Policies that influence the type and quality of food and beverages available in schools has the potential to reach most children within a country, especially where education is compulsory. Such food provisioning is therefore an opportunity to improve child growth and development for all children noting that poor diet quality in high-income countries exists regardless of a families’ socio-economic position. [14]. For children living with disadvantage who may be experiencing food and nutrition insecurity, SPMs are essential in alleviating hunger and ensuring children are given the opportunity to learn effectively [7]. In high-income countries with predominantly industrial dietary patterns SPMs have been touted as the potential “magic bullet” for childhood obesity or in some cases as the *“hero – rescuing children from the evils of the modern diet”* [15 p.1]. Japan is often used as the example, with one of the lowest rates of childhood obesity in the world (4%) the compulsory school lunch program (with a parent co-contribution) has been identified as one of the main contributing factors [16].

Most data on the effectiveness of SPMs comes from the USA and the introduction of the Healthy, Hunger-free Kids Act (HHKA), which implemented strengthened nutrition standards to SPMs (lunch, breakfast, canteens and vending machines) in 2012–2013. The introduction of the HHKA acted as a natural experiment moving schools to enforceable nutrition standards and the introduction of school-wellness policies. The HHKA has led to overall improvements in children’s diet quality [17]. Models of SPM provision in the USA vary from state to state. However, universal, free school meals in the USA appear to be effective in attenuating or reducing childhood obesity in all children with greater gains in children living in poverty [18–20]. A recent review concluded that universal, free school meals increased meal participation, and positively impacted diet quality and health among students regardless of household income [21]. Evidence from the review is less clear regarding the impact on weight status [21]. In England, the introduction of universal, free SPMs in the first three years of schooling (a switch from means-tested SPMs) has significantly reduced BMIz with bigger impacts for younger children [22, 23]. The reintroduction of free school meals in a South Korean province had a positive influence on overweight in adolescent boys and girls [24].

The effectiveness of SPMs on weight status within individual school environments can be masked by factors such as uptake of SPMs (while the food provisioning may be universal not all students may participate for a variety of reasons), and the availability of other options for food onsite, including bringing food from home, competitive foods (that is, foods not offered within the breakfast/lunch programs) for sale, vending machines and co-located food providers outside of the school grounds [25, 26]. Many schools concurrently offer competitive foods) for sale within the school environment – the quality of these foods may not be regulated and can compete with the SPMs reducing their uptake [27]. Marketing of energy-dense, nutrient-poor foods may also not be restricted sending mixed messages to children and young people. Only 28% of countries have policies that govern competitive food provisioning or marketing of foods within school environments [27]. There is also evidence that food brought from home as a “packed lunch” is of variable quality and costly (financial and time) for parents [28–30]. Failure to take these factors into account when evaluating the effectiveness of SPMs for individual children can lead to ambivalent results [21, 31, 32].

What becomes obvious in reviewing the effectiveness of SPMs on child weight status is that effectiveness is dependent on the model of provisioning (universal/targeted; free/subsidised/full payment) (see Table 1 for definitions) and whether SPMs is one element of a multicomponent approach. SPMs offered in conjunction with whole-of-school approaches that integrate with curriculum and provide opportunities for social engagement and critical skill development with compulsory physical education are potentially more effective [7, 33]. Framing SPMs as a strategy to address obesity is appealing as this outcome has a commonly understood and reported measure which connects the intervention to local, national and international monitoring and surveillance systems [34]. However, a sole focus on this outcome underrepresents the benefits of SPMs and is potentially a missed opportunity to address health more holistically [35].

**Table 1:** Definition of SPM implementation models

Universal	SPMs are provided to all children in all public schools irrespective of geographic or socio-economic location
Targeted	SPMs are provided to some children or to some schools based on certain criteria, usually related to the socio-economic status of the children
Free	Meals are provided free of charge to the parents/caregivers. There is no expectation that the household will pay
Subsidised/full payment	Some caregivers are expected/required to pay full-price for the meal; other households are eligible for subsidised meals usually based on means-testing

## SPMs are about more than Weight Status

Historically most SPMs were introduced to address malnutrition and undernourishment that emerged after global conflict with the goal to prevent hunger rather than necessarily focus on diet quality [1, 7]. As the global food system has developed there has been a shift in focus and many high-income countries have introduced nutrition standards to improve quality by providing more nutritious offerings. While obesity can be one driver for the implementation of SPMs, as discussed above, weight status is only one of many potential outcomes of nutritious SPMs. With the release of the *Lancet Diabetes and Endocrinology Commission* guidance on defining and diagnosing clinical obesity, it is important not to focus solely on weight outcomes (i.e. BMIz) [36]. This sole focus has further challenges when addressing weight-based bias and stigma and the need for public health strategies to move beyond individual responsibility [36]. In addition, conceptualizing SPMs using a human rights lens provides opportunities to consider children and young people as agentic beings with complex and diverse needs who live in dynamic environments [37]. For SPMs to be successful they need to focus beyond weight as a primary outcome and consider the overall social and psychological wellbeing of children and the communities in which they live(now and into the future), to provide skills to navigate increasingly complex environments and to be a vehicle for food system transformation.

Table 2 highlights features and objectives of selective SPM programs that are either successful or longstanding in high income countrieswith high rates of childhood obesity prevalence, or in the case of Japan where childhood obesity prevalence has been effectively minimised.

**Table 2:** Summary of the key features of SPM in selected high-income countries

**Country/Region**	Year SPM est*	**Model**	**Reach**	**Other components**	**Goal/Objective**
England	1945	Mixed Universal, free for infants (UFISM) (Reception, Yr1, Yr2) Universal, free in some disadvantaged areas Means-tested to be free in other areas	2.1 million children eligible for free school meals ~ 50% uptake 3.5 million children pay for meals [51]	• Procurement policies in place • Range of resources for leadership and curricula [52]	The School Food Plan is about “good food and happiness. It is about the pleasures of growing, cooking and eating proper food. It is also about improving the academic performance of our children and the health of our nation” [53 p.7]
Finland	1948	Universal Free	850,000 students from pre-primary to vocational education [54]. Only 10% of primary school students eat all components [55]. Uptake variable in secondary schools [56]	• Curriculum integration • Teachers required to eat with students • Guidelines for vending machines and competitive foods • Sustainability and public procurement principles are incorporated [56]	School meals that produce a joy of eating combine tasty, nutritious, sustainable, healthy and safe food with meal discussions related to food and sensory experiences as well as learning of a lifestyle that promotes well-being [57] For upper secondary and vocational students, the goal is to promote students’ ability to study and to enhance the lifelong health and wellbeing of every member of the school community while preventing diet-related illnesses [58]
France	1971	Universal Means-tested (*quotient familiale*) calculated yearly, based on household income and living expenses, and the family is charged accordingly [59] Participation voluntary	12 million children eligible, ~ 75% uptake [60]. Lower uptake for more disadvantaged students [61]	• Monitored by National Council of School Meals • Vending machines are prohibited • In 2025, plastic cooking and serving containers were banned [60]	• To meet educational goals • To provide a social safety net • To meet nutritional and/or health goals • To prevent or mitigate obesity [61] Limited data available. Meals served in schools potentially better quality than those served at home
Japan	1954	Universal Compulsory Low-income families can receive financial support. Schools responsible for the cost of provision, parents for the cost of ingredients	9 million children in primary and lower secondary (compulsory years of education) [62]	• Integrated with curriculum by law (Basic Act on Shokuiku—FNE) • Nutrition professionals in each school • Local procurement policies [62]	• To maintain and promote health through the intake of appropriate nutrition • To deepen a correct understanding of food in daily life, to cultivate the ability to make judgments that enable healthy eating habits, and to cultivate desirable eating habits • To enrich school life and cultivate a cheerful spirit of sociability and cooperation • To deepen the understanding that dietary habits are based on the blessings of nature, and to cultivate a spirit of respect for life and nature, as well as an attitude that contributes to the preservation of the environment • To deepen the understanding that dietary habits are supported by the various activities of people related to food, and to cultivate an attitude that values work • To deepen understanding of the excellent traditional food culture of Japan and other regions To lead to a correct understanding of the production, distribution and consumption of food [63]
Sweden	1946	Universal Free	2.1 million students, 100% of all primary and secondary school-aged children [1]	• Municipalities responsible for SPM [64] • Hot meal, vegetarian offering, salad bar, bread and beverages (water and milk) suggested daily [64] • Pupil-focused programs, equipped with skilled kitchen staff [64]	Free provision of meals to all students, that are: • tasty, • nutritious, • safe, • eco-smart, • pleasant and • integrated within the school, including in education [64]
USA	1946	Mixed Free for households with incomes at or below 130 percent of the Federal poverty level for their household size [65] Some states have introduced universal, free meals. Community Eligibility Provision applies if most children at a school would be eligible for free meals	29.6 million children (75% provided free or at reduced cost) [65]	• Schools can only use USDA produce and are encouraged to use local procurement [65]	As a measure of national security, to safeguard the health and well-being of the Nation’s children and to encourage the domestic consumption of nutritious agricultural commodities and other food, by assisting the States, through grants-in-aid and other means, in providing an adequate supply of foods and other facilities for the establishment, maintenance, operation, and expansion of nonprofit school lunch programs [66]

FNE- Food and Nutrition Education. Mixed—universal in some areas but not others, free in some schools but not all

*this year references the first known date of a nationally endorsed program. It is not the date for current iterations or updates

SPMs have been shown to have impacts for children, families, community and society. Impacts for students include improving dietary intake (such as increasing fruit, dairy, B vitamins, vitamin C, calcium intake, and reducing processed foods, sodium, saturated/total fat) and habits, behaviour, engagement in schooling [38–40]. Changes to diet quality will impact overall health, growth and development, and likely contribute to optimising weight status. Presence of SPMs nutrition standards are key to enhancing children’s dietary intake, through provision of fruits, vegetables and wholegrains [31]. Evaluation of the implementation of the Healthy, Hunger-Free Kids Act of 2010 in the USA, observed a 24% increase in maximum Healthy Eating Index 2010 scores, compared to pre-implementation [41]. A natural experiment evaluation of implementation of food and nutrient-based standards in the UK noted improvements in nutritional quality of lunches, which in turn meant children consuming SPM had higher intakes of protein, vitamin C and folate and lower percentage of energy from saturated fat, than children consuming packed lunches [42]. While benefits for children are often described as resulting from an increase in attendance when meals are provided, and therefore children are at school to engage in learning; a systematic review has noted there are mixed impacts on school attendance [21]. Similarly, another systematic review has found there are mixed impacts on academic performance (positive or no change) [31]. However, benefits on classroom behaviour and concentration have been noted [31, 43].

## SPMs Alleviate Household Food and Nutrition Insecurity

Benefits of SPMs (depending on the model) can extend to families including improving food security and equity. Trials of SPMs in countries (Norway, New Zealand) that were previously predominately ‘lunchbox systems’ have led to students, families, teachers and principals all perceiving positive changes to inequalities between students [43, 44]. Evaluation two years after the introduction of free SPMs in New Zealand noted the program reduced financial hardship/stress for families [44]. The role of SPMs in alleviating the parental burden of packing lunchboxes and their associated costs are consistently reported [9, 28, 45]. SPMs has been shown to reduce spending on groceries in low-income households in the USA [46]. It is logical that provision of SPMs will immediately alleviate hunger but the impact on household food and nutrition security more broadly requires further investigation.

## SPMs Build Relationships and Social Interactions

SPMs when implemented as acts of commensality can impact child well-being and the development of social competence [47]. According to sociocultural theory, children develop and construct understanding and meaning through social interaction with others, within their unique cultural contexts [48]. Mealtimes offer the opportunity for these interactions away from the constraints of sitting behind a desk in a classroom or outside in the playground. Using shared mealtimes to relax, discuss issues of importance and have fun, are highly valued by children and young people [47]. There is an opportunity to create a school food environment that encourages meals to be consumed socially, creating a sense of social belonging [67, 68]. Children participating in trials of SPMs have reported the value in sharing the same foods [69]. It is an immediate tangible shared experience where children bond over the quality of the food served. It is also seen by young people as a way break down social and health inequalities, whereby everyone eats the same thing, and no one goes without [43]. These benefits may extend beyond the mealtime itself and are reported to contribute to the development of more positive feelings by children towards school more broadly [69]. For example, following one and five years of a trial of SPMs in Norway, children and teachers described benefits in terms of children’s social skills with meals providing opportunities to make new friends and develop new skills such as table etiquette and conversation [43]. Many Swedish schools use the pedagogic meals approach, with meals used as an opportunity for social eating, with children and teachers eating together, supporting behavioural modelling, with meals considered part of informal education within the school day [67]. Further, it has been suggested that by offering students this opportunity for social learning and attendant social skills, shared mealtimes prepare young people to navigate life after school and successfully participate in their communities [70].

## SPMs have Broader Environmental, Community and Social Benefits

Schools are examples of contained food systems with linkages to the broader local, national and global food system. Food is procured, grown, prepared, distributed, sold and waste is disposed of. External and internal actors influence the types of food available and how they are consumed. Conceptualizing schools using food systems thinking highlights the links between schools, the local market and the broader food supply. It also highlights the opportunities for children to be considered as agentic actors who influence but are also influenced by this food system [72]. Collectively schools have substantial purchasing power and can be *“instruments of transformation, nudging markets towards more sustainable and equitable practices”* [1 p.11]. For those countries that have implemented SPMs there is a focus on improving food provision to reduce greenhouse gas emissions which includes the types of meals provided and minimising waste, for example in Sweden [8]. The bulk preparation of food for all children has the potential to reduce individual packaging found in food brought from home [73]. SPMs procurement policies have the potential to guide planetary health by minimising the length of the food supply chain. By prioritising local producers, SPMs can support local agricultural production and can trigger production diversification; therefore, enhancing local economies and supporting the implementation of sustainability procurement targets [8, 74]. For example, in Brazil at least 30% of federal funds go to produce supplied by local small-scale or family farms, similar targets could be adopted by SPMs in high income countries [50]. These programs are not without challenges and attention needs to be paid to adequate resourcing, infrastructure and attention to supply chains to ensure long-term viability [75].

SPMs have additional community and societal benefits including local employment, food systems and environmental impacts [49, 71]. There are gaps in the literature measuring several of the community and societal benefits, with most attention given to the more immediate, student focused outcomes.

## Whole School and Community Approach to SPMs

While SPMs are a key avenue in creating a health promoting school environment, international evidence indicates the impacts and outcomes achieved for children are optimised when meals are delivered within a comprehensive, multi-strategy school food system, or a ‘whole-school approach to food’. This approach includes creating a positive food environment (e.g. stigma free, culturally inclusive, safe), and integration of food and nutrition education. A whole-of-school approach with integrated curricula and experiential food literacy facilitates the development of children, as described by the Finnish SPMs enabling them to *“master the everyday food choices, the diversity of food alternatives and their significance, aiming at food competence and creation of a food sense”* [11 p.13] beyond the school boundaries.

A comprehensive approach is also adopted in longstanding, successful SPM programs, such as the programs nationally implemented in Sweden and Japan. Swedish SPMs prioritise meal enjoyment, social interaction and culture, and focus on addressing student welfare and societal equity, not weight status [67]. Integration of food education, supporting social learning, and the creation of positive food and eating environments within the SPMs system were described as key approaches to improve child development and behaviours as well as contribute to building food and sustainability knowledge [6]. Japan has, since 2005, mandated *Shokuiku* or food education in schools. The highlights of *Shokuiku* are that it; experientially develops an appreciation of food as integral to culture, tradition and its fundamental role in cultivating a deeper sense of human life and its interdependence with nature [76]. Creation of social mealtimes and positive eating environments are components described in recently published case studies of SPMs across four countries, demonstrating a growing recognition of the value of a comprehensive system to optimise health promotion opportunities [8].

The value of using a comprehensive, whole-school approach within the school setting is well-established. A review of international studies has found the use of appropriately resourced, multi-component whole-of-school approaches (including components addressing the environment, education, and social engagement) support healthy eating behaviours [32]. Single component, such as food provision alone, and multi-component initiatives may be equally effective on BMIz, fruit and vegetable intakes [77] but it is likely that comprehensive SPM programs can achieve wider wellbeing, behaviour and societal benefits [78–80]. As such, offering a comprehensive system integrating approaches beyond the nutrient value of the food on the plate, is integral in creating a health-promoting environment.

## Critical Elements for Successful SPMs

**Fig. 1:**
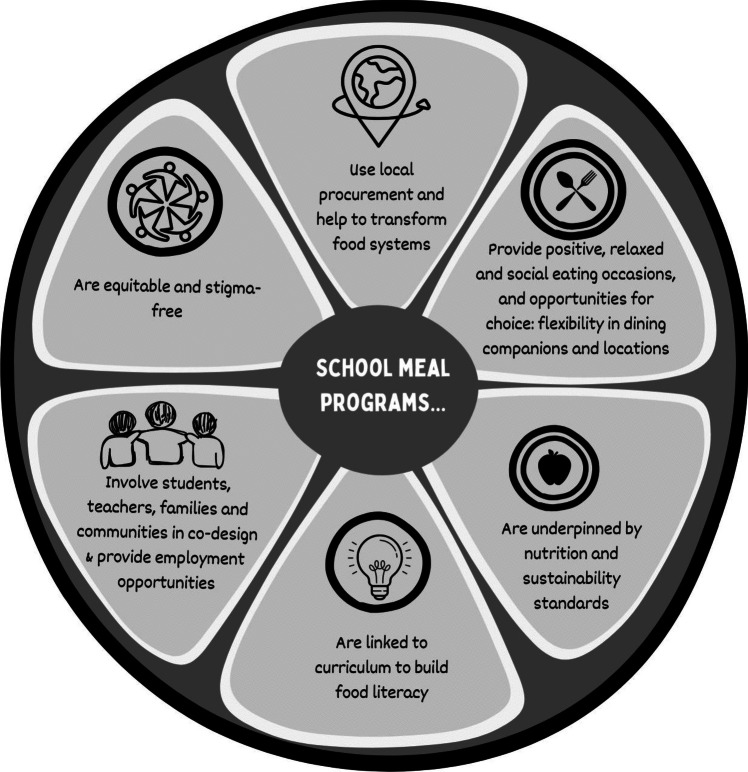
Critical elements for successful SPMs

In reviewing the impact of SPMs we identified the critical elements that will enhance their acceptability and effectiveness in influencing child, community and environmental health and wellbeing. These are summarised in Fig. 1. Based on our interpretations, it is key that SPMs are equitable so that families and children most in need can access them in a way that is stigma-free. In addition, SPMs need to: be underpinned by enforceable nutrition and sustainability standards; engage the local community to generate opportunities and enhance livelihoods; embed within a whole of school approach that conceptualises schools as transformative food systems; source foods locally used incentivised local procurement policies; integrate with curriculum that promotes lifelong food literacy capability development; provide occasions for commensality that celebrate culture and are positive and relaxed; and consider opportunities for delivery outside of regular school hours and during emergencies.

## Strengths, Weaknesses and Recommendations for Future Research

This was not a systematic review of the literature and did not include formal critical appraisal of publication bias however, several recent systematic reviews were drawn on and a review of grey literature and peer-reviewed literature was undertaken to broaden the perspectives beyond the USA. We do note however, that there may be reports published in languages other than English that we were unable to access. The strength of this review is that it attempted to go beyond individual benefits to explore the role of SPMs in generating more upstream influences which ultimately indirectly impact on child health and wellbeing. Most papers focus on the impact of SPMs at the country level and as such benefits at the school-level may be under-reported. However, not all countries are publishing the impact of SPMs and in countries where they are culturally accepted as the norm, their impact is under-reported. What is noticeable is that very few SPM programs are underpinned by evaluation frameworks and logic models that explicate their potential role in holistic child, family and community health and wellbeing. Future research needs to explore robust process, impact and outcome evaluation that provide data to enhance routine implementation and the benefits of SPM beyond the individual.

## Conclusion

SPMs have the potential—if part of a comprehensive program—to influence children’s weight status via multiple pathways. The provision of the meal itself can directly impact on physical wellbeing, through improvements in diet quality. However, a more holistic approach could impact on children’s health, by influencing social and cultural wellbeing and a sense of belonging, improving food and nutrition security, enhancing local food systems and economies, providing food literacy skills that empower navigation of food systems and transforming food systems towards sustainability. A sole focus on weight status outcomes could do SPMs a disservice as they have the potential to provide so much more, not only for children but for families and communities. SPMs are an important universal basic need for the social good. If implemented with a focus on the holistic child, they have the promise to build critical life skills that enables navigation of food environments irrespective of context and to support the communities and environments in which children live.

## Key References

- Manson, A. C., Golley, R. K., & Johnson, B. J. Global parent perspectives on school food service internationally: A mixed papers narrative review. Nutr Diet, Online. 2025; https://doi.org/10.1111/1747-0080.12926.
  o Showcases different models of SPMs each incorporating several of the critical elements for successful SPMs.
- Kerr, J. A., Patton, G. C., Cini, K. I., Abate, Y. H., Abbas, N., Abd Al Magied, A. H. A., et al. Global, regional, and national prevalence of child and adolescent overweight and obesity, 1990–2021, with forecasts to 2050: a forecasting study for the Global Burden of Disease Study 2021. Lancet. 2025;405(10,481), 785–812, https://doi.org/10.1016/S0140-6736(25)00397–6.
  o Provides the latest international data on overweight and obesity prevalence including forecasting to 2050, highlighting the ongoing importance of obesity.
- Swinburn, B. A., Kraak, V. I., Allender, S., Atkins, V. J., Baker, P. I., Bogard, J. R., et al. The global syndemic of obesity, undernutrition, and climate change: The Lancet Commission report. Lancet. 2019;393(10,173), 791–846, https://doi.org/10.1016/S0140-6736(18)32,822–8.
  o The pandemics of obesity, undernutrition and climate change are simultaneously impacting each other at the same time, place and within populations across the globe.
- Research Consortium for School Health and Nutrition. School Meals and Food Systems: Rethinking the Consequences for Climate, Environment, Biodiversity, and Food Sovereignty; 2023. [March 17, 2025] Available from: https://schoolmealscoalition.org/sites/default/files/2024-09/Pastorino_etal_JAN_2024_School-meals-and-food-systems%20%281%29.pdf
  o White paper by the School Meals Coalition that highlights the importance of considering how SPMs can impact planetary health and food sovereignty.
- Rappleye, J., Komatsu, H., & Nishiyama, S. School food, sustainability, and interdependence: learning from Japan’s Shokuiku? Oxford Rev Educ. 2025;51(1), 129–147, 10.1080/03054985.2023.2296097.
  o Provides an example of how several of the critical elements of successful SPMs could be integrated through Shokuiku.

## Data Availability

No datasets were generated or analysed during the current study.
